# SCF Framework, HF Stability, and RPA Correlation for Jordan–Wigner-Transformed Spin Hamiltonians on Arbitrary Coupling Topologies

**DOI:** 10.1021/acs.jctc.6c00095

**Published:** 2026-04-24

**Authors:** Shadan Ghassemi Tabrizi, Thomas M. Henderson, Thomas D. Kühne, Gustavo E. Scuseria

**Affiliations:** † Computational System Sciences, Technische Universität Dresden, 01187 Dresden, Germany; ‡ Center for Advanced Systems Understanding (CASUS), Am Untermarkt 20, 02826 Görlitz, Germany; § Department of Physics and Astronomy, 3990 Rice University, Houston, Texas 77005-1892, United States; ∥ Department of Chemistry, 3990 Rice University, Houston, Texas 77005-1892, United States

## Abstract

Mapping spins to fermions via the Jordan–Wigner (JW) transformation can render mean-field (Hartree–Fock, HF) descriptions effective for strongly correlated spin systems. As established in recent work, the application of such approaches is not limited by the nonlocal structure of JW strings or by site ordering because string operators can be absorbed into Thouless rotations of a Slater determinant, and the variational optimization of a unitary Lie-algebraic similarity transformation removes any ordering dependence. Leveraging these ideas, we develop a self-consistent field (SCF) scheme that expresses the mean-field energy as a functional of the single-particle density matrix, providing an alternative to gradient-based optimization of Thouless parameters. We derive the analytical orbital Hessian to diagnose HF stability and compute the ground-state correlation energy through the random-phase approximation (RPA). Benchmark results for the *XXZ* and *J*_1_–*J*_2_ model on one- and two-dimensional lattices demonstrate that RPA significantly improves mean-field accuracy.

## Graphical abstract

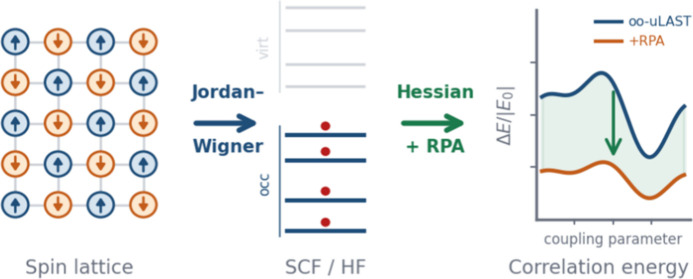

## 1 Introduction

The physical properties of exchange-coupled spin clusters (e.g., magnetic molecules or lattice fragments of quantum magnets) are commonly modeled with spin Hamiltonians^1,2^ such as the Heisenberg model, *H* = ∑_*m*<*n*_ *J*_*mn*_ **s** *_m_* ·**s** *_n_*. Although the use of spin and point-group symmetries to factor the Hamiltonian into invariant subspaces^3–5^ extends the reach of exact diagonalization (ED), the exponential growth of the Hilbert space dimension with the number of sites (2*^N^* for *N* spin-1/2 sites) still confines exact calculations to relatively small lattices. A wide range of approximation schemes—e.g., density matrix renormalization group^6,7^ (DMRG) and other tensor-network methods,^8^ quantum Monte Carlo,^9^ coupled-cluster theory,^10–12^ or spin-wave expansions^13^—address complementary regimes of quantum magnets with different computational costs, accuracies, and accessible observables. A comprehensive review is beyond the scope of this work.

Against this landscape, mean-field ideas^14–16^ remain attractive for their conceptual transparency, low computational cost, and broad applicability. In earlier work,^16^ we implemented efficient mean-field treatments of JW-transformed spin Hamiltonians and captured correlation via Lie-algebraic similarity transformations^15,17^ (LAST). Here, we recast the Hartree–Fock (HF) problem in an SCF formulation familiar with quantum chemistry. We express the mean-field energy as a functional of the single-particle density matrix **ρ**, interpret JW strings as Thouless rotations,^14^ and remove site-ordering dependence via unitary LAST (combined with HF, this constitutes the orbital-optimized uLAST method,^15^ oo-uLAST, see Section 2). The SCF formulation allows to leverage standard convergence machinery (damping, level shifting, DIIS, etc., see, e.g., ref 18 and references cited therein), provides stability diagnostics via the analytic orbital Hessian^19–21^ derived here (the examination of Thouless’ stability conditions in a quantum-chemical context was pioneered by Čížek and Paldus^22,23^ ), and interfaces naturally with response theory, specifically the random-phase approximation (RPA) for correlation energies^24–27^ on top of the oo-uLAST reference. This yields a scalable mean-field framework for ground states of spin Hamiltonians with arbitrary coupling topologies, as encountered, e.g., in Heisenberg-type models of polynuclear transition-metal complexes and active sites of metalloenzymes, complementing the nonunitary LAST correlation approach from recent work.^15,16^

In Section 2, we first briefly recapitulate the JW transformation,^28^ with emphasis on its extended variant^29,30^ (EJW). The uLAST ansatz amounts to a variational optimization of the EJW parameters.^15^ We then formulate the energy of a Slater determinant in the EJW fermionic representation as a functional of **ρ** and derive the associated Fock matrix to be employed in an SCF scheme. Next, we construct the orbital Hessian and the closely related RPA matrix. Their spectra provide stability diagnostics of stationary HF points (distinguishing minima from saddle points) and access to ground-state correlation energies with respect to oo-uLAST, where we employ the ring-CCD convention^25,31^ (prefactor $\frac{1}{4}$ in eq 38 below) for exchange-including kernels.

Section 3 examines a set of one- and two-dimensional lattices, including various parameter regimes of the *XXZ* and antiferromagnetic *J*_1_–*J*_2_ models. Appendix A provides an alternative formulation of the uLAST gradient and derivations of the Fock kernel and the **A** and **B** matrices required to construct the orbital-Hessian and RPA matrices.

## 2 Theory

### 2.1 Extended JW and uLAST

The (extended) JW mapping of spins to fermions,

$$s_{p}^{+}=c_{p}^{†}\phi_{p}^{†}$$ 1

$$s_{p}^{-}=c_{p}\phi_{p}$$ 2

$$s_{p}^{z}=n_{p}-\frac{1}{2}$$ 3

expresses the spin ladder operators at site *p*, *s*_*p*_^+^ = *s*_*p*_^*x*^ + *is*_*p*_^*y*^ and *s*_*p*_^–^ = *s*_*p*_^*x*^ – *is*_*p*_^*y*^ in eqs 1 and 2, respectively, in terms of fermionic creation and annihilation operators, *c*_*p*_^†^ and *c*_*p*_. Up to a constant shift, the *z*-component of the local spin, *s*_*p*_^*z*^, corresponds to the occupation number of the respective orbital, *n*_*p*_ = *c*_*p*_^†^ *c*_*p*_. The correct commutation (anticommutation) relations for spins (fermions) are afforded by string operators, eq 4, parametrized by real angles θ_*pq*_, under the constraints θ_*pq*_ = θ_*qp*_ + π for *p* < *q*, and θ_*pp*_ = 0.

$$\phi_{p}^{†}=e^{i\sum_{q}\theta_{pq}n_{q}}$$ 4

The conventional JW transformation is a special case in which θ_*pq*_ = 0 for *q* < *p*, and we denote the corresponding fermionic Hamiltonian by *H*_JW_. The more general *H*_EJW_ is obtained by a unitary Lie-algebraic similarity transformation (uLAST) of *H*_JW_ (eq 5),

$$H_{\mathrm{EJW}}=e^{-\gamma }H_{\mathrm{JW}}e^{\gamma }$$ 5

by the two-body correlator of eq 6:

$$\gamma =\frac{1}{2}\underset{p,q}{\sum }\gamma_{pq}n_{p}n_{q}$$ 6

where γ_*pq*_ = *i*Θ*_pq_*, with a real symmetric matrix **Θ**; the strings occurring in *H*_EJW_ (cf. eq 4) are then defined by angles θ_*pq*_ = Θ*_pq_* for *p* < *q* and θ_*pq*_ = Θ*_pq_* + π for *p* > *q*. Treating EJW angles θ_*pq*_ as optimization parameters makes the uLAST ansatz independent of the site numbering.

### 2.2 SCF Formulation

Within the oo-uLAST framework, we variationally optimize both the free EJW parameters (corresponding to a uLAST approach) and a Slater determinant for the EJW-transformed Hamiltonian (this HF part represents the orbital optimization). In our recent work, we showed how to optimize uLAST parameters by gradient descent simultaneously with orbital optimization.^16^ In the Appendix of the present paper, we derive an alternative uLAST gradient evaluated at an HF stationary point, which is expressed in terms of **ρ** and does not reference the HF wave function. Together with the SCF formulation introduced below, this density matrix form could support schemes akin to finite-temperature (thermal) HF,^32^ though we do not pursue that direction here.

In what follows, we cast the HF problem as an SCF procedure. With properly defined values for one- and two-particle integrals, *t*_*kl*_ and [*kn* | *lm*], the *z*-coupling contains no strings and can be written in the standard second-quantized form of eq 7, with a constant contribution $E_{\mathrm{const}}=\frac{1}{4}\underset{m<n}{\sum }J_{mn}$ .

$$\underset{m<n}{\sum }J_{mn}s_{m}^{z}s_{n}^{z}=E_{\mathrm{const}}+\underset{kl}{\sum }t_{kl}c_{k}^{†}c_{l}+\frac{1}{2}\underset{klmn}{\sum }\left[kn|lm\right]c_{k}^{†}c_{l}^{†}c_{m}c_{n}$$ 7

Thus, a Fock matrix **F** *^z^* for *z*-coupling can be constructed from the single-particle density matrix, ρ_*kl*_ ≡ ⟨Φ|*c*_*l*_^†^ *c*_*k*_|Φ⟩, in the usual way (see eq 8),

$$F^{z}=t+\Gamma$$ 8

with **Γ** defined in eq 9:

$$\Gamma_{kl}=\underset{mn}{\sum }\left[kl|mn\right]\rho_{nm}$$ 9

The respective contribution to the mean-field energy is given in eq 10:

$$E^{z}\left[\rho \right]=\mathrm{Tr}\left(t\rho \right)+\frac{1}{2}\mathrm{Tr}\left(\Gamma \rho \right)+E_{\mathrm{const}}$$ 10

In contrast, *xy*-coupling involves string operators (strings vanish in open chains with nearest-neighbor interactions; in rings, a remaining string for the coupling between the first and last sites reduces to a sign factor in a definite fermion-number sector). For a pair ⟨*m*, *n*⟩, we can pull the string operators to the right (eq 11),

$$s_{m}^{x}s_{n}^{x}+s_{m}^{y}s_{n}^{y}=\frac{1}{2}s_{m}^{+}s_{n}^{-}+h.c.=\frac{1}{2}\left[c_{m}^{†}c_{n}\phi_{m\left(n\right)}^{†}\phi_{n\left(m\right)}+h.c.\right]$$ 11

by defining reduced strings, ϕ_*m*(*n*)_^†^ = *e* ^*i*∑_*q* ≠ *n*_θ_*mq*_ *n*_*q*_^. The insight that strings act on Slater determinants as Thouless rotations^14^ has recently enabled an efficient implementation of mean-field and subsequent correlation methods.^16^ The unitary orbital rotation $R\in C^{N_{\mathrm{orb}}\times N_{\mathrm{orb}}}$ effected by a general string *e* ^*i*α_q_ *n*_q_^ is given in eq 1, where $C\in C^{N_{\mathrm{orb}}\times N_{f}}$ and **C** *_R_* collect the orthonormal occupied orbitals defining the Slater determinants |Φ⟩ and |Φ*_R_* ⟩ ≡ *e* ^*i*∑_*q*_α_*q*_ *n*_*q*_^, respectively; *N*_orb_ is the size of the single-particle basis, and *N*_f_ is the fermion number.

$$C_{R}=\mathrm{RC}=\left(\begin{array}{ccc} e^{i\alpha_{1}} & 0 & 0\\ 0 & \ddots & 0\\ 0 & 0 & e^{i\alpha_{N_{\mathrm{orb}}}} \end{array}\right)C$$ 12

With the aim of deriving the *xy*-coupling contribution to the Fock operator, **F** *^xy^*, we now formulate the respective mean-field energy as a functional of the single-particle density matrix, **ρ** = **CC** ^†^. Equation 13 for the overlap between the original and the rotated determinant invokes the overlap matrix **S** ≡ **C** ^†^ **C** *_R_*.^33^

$$w\equiv \left\langle \Phi \right|\Phi_{R}\rangle =\det\left(S\right)$$ 13

To express *w* as a functional of **ρ**, we introduce auxiliary matrix **Z** in eq 14, where **1** is the unit matrix.

Z=1+ρ(R−1) 14

Using Sylvester’s matrix-determinant identity (eq 15),

det(1+LM)=det(1+ML) 15

and setting **L** = **C** and **M** = **C** ^†^(**R** – **1**), we obtain eq 16:

$$\det\left(Z\right)=\det\left[1+{\mathrm{CC}}^{†}\left(R-1\right)\right]=\det\left[1+C^{†}\left(R-1\right)C\right]=\det\left(S\right)=w$$ 16

The single-particle transition-density matrix **ρ** *_R_* is defined in eq 17:

$${\left(\rho_{R}\right)}_{kl}=\frac{\left\langle \Phi |c_{l}^{†}c_{k}\right|\Phi_{R}\rangle }{\left\langle \Phi \right|\Phi_{R}\rangle }$$ 17

Equation 18 is a standard result from a generalized Wick’s theorem (see, e.g., ref 33, and references cited therein):

$$\rho_{R}={\mathrm{RCS}}^{-1}C^{†}$$ 18

In order to recast **ρ** *_R_* as a density matrix functional, we write eq 19,

$$\begin{array}{l} {\mathrm{ZCS}}^{-1}C^{†}=\left(1+{\mathrm{CC}}^{†}R-{\mathrm{CC}}^{†}\right){\mathrm{CS}}^{-1}C^{†}\\ ={\mathrm{CC}}^{†}{\mathrm{RCS}}^{-1}C^{†}+\left(1-{\mathrm{CC}}^{†}\right){\mathrm{CS}}^{-1}C^{†}=\rho +0 \end{array}$$ 19

hence:

$$Z^{-1}\rho ={\mathrm{CS}}^{-1}C^{†}$$ 20

Combining eqs 20 and 18 then yields eq 21:

$$\rho_{R}={\mathrm{RZ}}^{-1}\rho$$ 21

Equation 22 gives the energy contribution from *xy*-coupling for a pair ⟨*m*, *n*⟩,

$$E_{mn}^{xy}=\left\langle \Phi \right|s_{m}^{x}s_{n}^{x}+s_{m}^{y}s_{n}^{y}\left|\Phi \right\rangle =\frac{1}{2}\left\langle \Phi \right|c_{m}^{†}c_{n}\phi_{m\left(n\right)}^{†}\phi_{n\left(m\right)}+h.c.\left|\Phi \right\rangle =w_{mn}\mathrm{Tr}\left(h_{mn}\rho_{R}^{mn}\right)+c.c$$ 22

where ${\left(h_{mn}\right)}_{ij}=\frac{1}{2}J_{mn}\delta_{im}\delta_{jn}$ ; *w*_*mn*_ and **ρ**_*R*_^*mn*^ are calculated with respect to the rotated Slater determinant |Φ_*R*_⟩ = ϕ_*m*(*n*)_^†^ϕ_*n*(*m*)_|Φ⟩.

The Fock matrix represents the functional derivative **ρ**; in terms of differentials, d*E* = Tr­(**F**d**ρ**). **F** is a sum of contributions from *z*-coupling (eq 8), and *xy*-coupling, **F** = **F** *^z^* + **F** *^xy^*. To obtain **F** *^xy^*, we consider the first term on the right-hand side of eq 22, i.e., the incremental energy contribution ε_*mn*_ = *w*_*mn*_Tr­(**h**_*mn*_ **ρ**_*R*_^*mn*^). We extract the respective Fock increment **f** *_mn_* from dε*_mn_* = Tr­(**f**_*mn*_d**ρ**). In the following derivation— leading to eq 28—to avoid clutter, we write ε, *w*, **h**, **ρ**_R_, and **f** instead of ε*_mn_*, *w*_*mn*_, **h** *_mn_*, **ρ**_*R*_^mn^, and **f** *_mn_*, respectively. The differentials for **Z** and **Z** ^–1^ are given in eqs 23 and 24, respectively,

dZ=(dρ)(R−1) 23

$$d(Z^{-1})=-Z^{-1}(dZ)Z^{-1}=-Z^{-1}(d\rho )(R-1)Z^{-1}$$ 24

thus:

$$d\rho_{R}=Rd(Z^{-1})\rho +{\mathrm{RZ}}^{-1}d\rho =-{\mathrm{RZ}}^{-1}(d\rho )(R-1)Z^{-1}\rho +{\mathrm{RZ}}^{-1}d\rho$$ 25

For the differential of the overlap (eq 26), Jacobi’s determinant formula and eq 23 are employed,

$$dw=d\;\det(Z)=\det(Z)\mathrm{Tr}(Z^{-1}dZ)=w\mathrm{Tr}[Z^{-1}(d\rho )(R-1)]$$ 26

which yields the energy differential in eq 27.

$$d\varepsilon =w\mathrm{Tr}(hd\rho_{R})+dw\mathrm{Tr}(h\rho_{R})=w\mathrm{Tr}({\mathrm{hRZ}}^{-1}d\rho )-w\mathrm{Tr}[{\mathrm{hRZ}}^{-1}(d\rho )(R-1)Z^{-1}\rho ]+w\mathrm{Tr}[Z^{-1}(d\rho )(R-1)]\mathrm{Tr}(h\rho_{R})$$ 27

Using the cyclicity of the trace to collect every occurrence of d**ρ** on the right, we finally obtain the Fock increment (eq 28),

$$f=w\left[{\mathrm{hRZ}}^{-1}-\left(R-1\right)Z^{-1}{\rho \mathrm{hRZ}}^{-1}+\mathrm{Tr}\left({h\rho }_{R}\right)\left(R-1\right)Z^{-1}\right]$$ 28

**F** *^xy^* represents the sum of increments for all ordered pairs (eq 29),

$$F^{xy}=\underset{m\neq n}{\sum }f_{mn}$$ 29

The usual SCF process consists in the following steps: (i) build the Fock matrix **F** = **F** *^z^* + **F** *^xy^* for some initial guess **ρ**, (ii) diagonalize **F**, i.e., **Fv** *_i_* = ε_*i*_ **v** *_i_*, yielding orbital energies ε_*i*_ and canonical orbitals **v** *_i_* (molecular orbitals, MOs, in quantum-chemical terminology), (iii) occupy the orbitals following the aufbau principle to form a new density matrix, **ρ** = ∑_*p* = 1_^*N*_f_^ **v**_*p*_ **v**_*p*_^†^, (iv) repeat until self-consistency is reached, where [**F**, **ρ**] = 0. Standard techniques for SCF acceleration can be employed. Note that even if one proceeds with a Thouless-parameter optimization rather than an SCF process, access to canonical orbitals and orbital energies is still valuable because it supplies a diagonal preconditioner for the gradient. As an example, rescaling the virtual-occupied blocks by (Δε_*vo*_)^−1/2^, where Δε_*vo*_ = ε_*v*_ – ε_*o*_ is the respective orbital-energy difference, was suggested in ref 34 to accelerate convergence.

The dominant cost of mean-field calculations on general coupling graphs comes from the *xy*-coupling terms, whose strings act as orbital rotations on a Slater determinant (see eq 12). As explained, evaluating the energy/Fock contributions for an interacting pair ⟨*m*, *n*⟩ requires overlaps and transition densities between |Φ⟩ and a string-rotated determinant |Φ*_R_* ⟩. The formal leading cost of building **f** *_mn_* scales as $O\left(N_{\mathrm{orb}}^{3}\right)$ , with *N*_orb_ being the number of spin-1/2 sites (or spin-1/2 auxiliaries representing $s>\frac{1}{2}$ sites, see below). The number of interacting pairs is typically $O\left(N_{\mathrm{orb}}\right)$ , implying an overall $O\left(N_{\mathrm{orb}}^{4}\right)$ scaling for a full Fock build; the subsequent diagonalization of **F** scales as $O\left(N_{\mathrm{orb}}^{3}\right)$ . In the Thouless-parameter optimization route,^16^ the mean-field state is parametrized by complex amplitudes *Z*_*vo*_. Each optimizer step requires an evaluation of the energy and its analytic gradient, which involves the same string-rotated overlaps/transition densities as above, hence the same $O\left(N_{\mathrm{orb}}^{4}\right)$ leading cost; an additional transformation from local to global gradient representations is an $O\left(N_{\mathrm{orb}}^{3}\right)$ step in the standard implementation.

We optimize the mean-field state and the uLAST parameters in a coupled fashion using a nested (macro/micro) procedure. In each macroiteration, the mean-field state is updated for fixed uLAST parameters by solving the SCF equations to (near‑)​stationarity, after which the uLAST parameters are updated using a gradient-based step evaluated at the (approximately) stationary mean-field solution. We accelerate SCF convergence using the OpenOrbitalOptimizer (OOO) package^18^ of Lehtola and Burns, a lightweight SCF driver providing standard stabilization and convergence-acceleration schemes (e.g., damping/level shifting and DIIS-type extrapolation). OOO is connected to our MATLAB code through a small C++/MEX interface layer that implements a callback: given the current orbital coefficients and occupations proposed by OOO, the interface constructs the (generally complex) density matrix **ρ** = **CC** ^†^ and calls a MATLAB routine to evaluate the HF energy, *E*[**ρ**], and the Fock matrix, **F**[**ρ**]. These quantities are returned to OOO, which performs the diagonalization and updates the orbitals using its built-in acceleration strategy. This cycle is repeated until self-consistency is reached. For robustness, we first perform a short preconditioning stage with our original MATLAB SCF loop using simple density damping and then start OOO from the corresponding Fock matrix.

### 2.3 Gauge Freedom in oo-uLAST

We note a redundancy in the parametrization of the real symmetric matrix **Θ** that defines the uLAST correlator γ (cf. eq 6). Consider the separable shift of eq 30,

$$\Theta_{pq}\to \Theta_{pq}^{\prime }=\Theta_{pq}+\chi_{p}+\chi_{q},\chi_{p}\in R$$ 30

which changes the generator Δγ = γ­(**Θ** ^′^) – γ­(**Θ**),

$$\Delta \gamma =\frac{i}{2}\sum_{p<q}(\chi_{p}+\chi_{q})n_{p}n_{q}=\frac{i}{2}\sum_{p}\chi_{p}n_{p}\sum_{q\neq p}n_{q}=\frac{i}{2}\sum_{p}\chi_{p}n_{p}(N-1)$$ 31

where $N\equiv \underset{q}{\sum }n_{q}$ . Within a fixed particle-number sector, $N=N_{f}$ , eq 31 reduces to a purely one-body operator, eq 32,

$$\Delta \gamma =i\underset{p}{\sum }\kappa_{p}n_{p},\kappa_{p}=\frac{1}{2}\left(N_{f}-1\right)\chi_{p}$$ 32

Since γ­(**Θ**) and Δγ commute, [γ­(**Θ**), Δγ] = 0, the shifted Hamiltonian is related to the original one by a unitary similarity transformation, eq 33,

$$H_{\mathrm{EJW}}\left(\Theta^{\prime }\right)=e^{-\gamma \left(\Theta^{\prime }\right)}H_{\mathrm{JW}}e^{\gamma \left(\Theta^{\prime }\right)}=U_{\chi }^{†}H_{\mathrm{EJW}}\left(\Theta \right)U_{\chi }$$ 33

where *U*_χ_ ≡ *e* ^Δγ^ represents a local U(1) transformation of the fermionic modes (eqs 34 and 35):

$$U_{\chi }^{†}c_{p}U_{\chi }=e^{i\kappa_{p}}c_{p}$$ 34

$$U_{\chi }^{†}c_{p}^{†}U_{\chi }=e^{-i\kappa_{p}}c_{p}^{†}$$ 35

Thus, within a fixed particle-number sector, the shift in eq 30 leaves the spectrum and all number-conserving observables invariant. This is a gauge redundancy of the parametrization that manifests in flat directions in the **Θ** parameter space. A simple gauge fixing is obtained by choosing a reference index *r* and imposing Θ*_rq_* = Θ*_qr_* = 0 for all *q*, which removes *N*_orb_ – 1 redundant degrees of freedom and thereby reduces the number of independent uLAST parameters from $\frac{1}{2}N_{\mathrm{orb}}\left(N_{\mathrm{orb}}-1\right)$ to $\frac{1}{2}\left(N_{\mathrm{orb}}-1\right)\left(N_{\mathrm{orb}}-2\right)$ . However, we found that the described gauge fixing frequently worsened the convergence properties of oo-uLAST calculations, and we therefore retained the redundant parametrization in the production calculations.

### 2.4 HF Stability and RPA

To assess the character of a stationary HF solution, we analyze the orbital Hessian, which defines the second derivative of the HF energy with respect to particle-hole (Thouless) rotations; see eq 36. In the particle-hole basis, it has block form:

$$H=\left(\begin{array}{cc} A & B\\ B^{*} & A^{*} \end{array}\right)$$ 36

A positive-definite **H** certifies HF stability^20,23^ (a local minimum), broken continuous one-body symmetries cause zero modes, and any negative eigenvalue signals an instability (a corresponding eigenvector provides a direction that can be used to distort the Slater determinant toward lower energy^35^). RPA, on the other hand, leads to an eigenproblem for the matrix **R** of eq 37,

$$R=\left(\begin{array}{cc} A & B\\ -B^{*} & -A^{*} \end{array}\right)$$ 37

which is constructed from the same blocks but with a different metric.^36,37^ For a positive-definite orbital Hessian, the RPA spectrum is real (conversely, a real RPA spectrum is not sufficient to guarantee positive-definiteness of the orbital Hessian^38,39^ ).

For a stable HF solution, the eigenvalues of **R** occur in real pairs ± ω, and these RPA eigenmodes can be used to estimate ground-state correlation energies. We evaluate the RPA correlation energy *E*_c_^RPA^ in the ring-CCD convention (eq 38), for a oo-uLAST reference via the summed difference between the spectra of RPA and TDA (Tamm–Dancoff approximation, setting **B** = 0 in the RPA matrix, eigenvalues ± ν), considering only the positive RPA and TDA eigenvalues:

$$E_{c}^{\mathrm{RPA}}=\frac{1}{4}\sum_{i}(\omega_{i}-\nu_{i})=\frac{1}{4}\left[\sum_{i}\omega_{i}-\mathrm{Tr}(A)\right]$$ 38

This choice is consistent with interpreting our exchange-including JW kernel as a fermionic ring-CCD resummation,^25^ whereas the plasmon formula^24^ would assign a prefactor of $\frac{1}{2}$ to the same spectrum, thus yielding correlation energies larger by a factor of 2.

Due to the nonlocal JW string operators, the fermionic Hamiltonian is not restricted to the usual one- and two-body terms. As a result, existing derivations of the orbital Hessian or the RPA matrix^36,37^ may not transfer directly to this setting. We therefore provide a self-contained, transparent derivation of **A** and **B** explicitly from the Fock kernel **K** in Appendix A2; **K** is the derivative of **F** with respect to **ρ** (see Appendix A3).

### 2.5 Larger Local Spins

To treat on-site spins $s>\frac{1}{2}$ within the JW framework, we represent each physical spin at site *p* by 2*s*_*p*_ auxiliary spin-1/2 degrees of freedom {**κ**_*p*, *a*_} (eq 39),

$$s_{p}\to \overset{2s_{p}}{\underset{a=1}{\sum }}\kappa_{p,a}$$ 39

and apply the JW (or EJW) mapping to these auxiliaries. Although this enlarges the Hilbert space, because the {**κ**_*p*, *a*_} can couple to local spins smaller than *s*_*p*_, the construction is variationally safe: As shown in our previous work,^16^ for bilinear spin Hamiltonians, the exact ground state has maximal local spin at every site, and, when *S*_*z*_ is conserved, the same holds within each *S*_*z*_ sector (with definite magnetic quantum number *M*). Thus, any trial state in the enlarged space provides an upper bound to the exact ground-state energy without requiring projection onto the maximal local spin subspace. Adopting the auxiliary spin-1/2 representation allows us to use exactly the same methodology (oo-uLAST within the SCF formulation and the subsequent RPA treatment of correlation) for systems with $s>\frac{1}{2}$ sites, also with site-dependent *s*, although our examples focus on uniform local spin.

## 3 Results and Discussion

We assess the practical performance of evaluating the RPA correlation energy on top of oo-uLAST references based on the proposed SCF/HF framework for a small set of benchmark systems. Using exact diagonalization (ED, in the uncoupled *S*_*z*_ basis, |*m*_1_, *m*_2_, ···, *m*_*N*_⟩, restricted to the *M* = 0 subspace) as a reference, we consider one- and two-dimensional coupling topologies in representative parameter regimes of the anisotropic *XXZ* model (eq 40),

$$H=\underset{\left\langle m,n\right\rangle }{\sum }\left(s_{m}^{x}s_{n}^{x}+s_{m}^{y}s_{n}^{y}+\Delta s_{m}^{z}s_{n}^{z}\right)$$ 40

and the isotropic *J*_1_–*J*_2_ model (eq 41),

$$H=J_{1}\underset{\left\langle m,n\right\rangle }{\sum }s_{m}\cdot s_{n}+J_{2}\underset{\left\langle \left\langle m,n\right\rangle \right\rangle }{\sum }s_{m}\cdot s_{n}$$ 41

which includes antiferromagnetic nearest-neighbor (NN, *J*_1_ > 0) and next-nearest-neighbor couplings (NNN, *J*_2_ > 0). All RPA energies are computed with respect to the converged oo-uLAST reference in the *M* = 0 sector, corresponding to half-filling *N*_f_ = *N*_orb_/2 in the fermionic picture. HF stability is assessed from the eigenvalue spectrum of the corresponding orbital Hessian, and we illustrate how the orbital-Hessian spectrum provides a diagnostic of the quality of the mean-field reference.

### 3.1 *XXZ* Model

Figure 1a compares relative ground-state energy errors for an *N* = 8 spin-1/2 *XXZ* chain as a function of the anisotropy Δ, using four levels of approximation: JW-HF, JW-HF+RPA, oo-uLAST, and oo-uLAST+RPA. Adding the RPA correlation systematically improves energies in most of the parameter range, with the largest impact in regions where the underlying reference is the least accurate. The orbital-Hessian spectrum for oo-uLAST plotted in Figure 1b explains the kink in the RPA energy curve at the isotropic point Δ = 1 in terms of an eigenvalue approaching zero, indicating a change in the character of the stationary mean-field reference along the scan (i.e., competing solutions becoming nearly degenerate). Consistent with earlier observations for one-dimensional systems,^15,16^ in the sequential numbering of sites along the chain, JW-HF and oo-uLAST are equivalent for Δ ≥ 0, whereas for Δ < 0, oo-uLAST affords lower variational energies than JW-HF, and the corresponding oo-uLAST+RPA curve provides the best overall description in this regime. In the following, we restrict our discussion to oo-uLAST references, because the numbering dependence^15^ and the performance of JW-HF for fixed site orderings relative to oo-uLAST have already been illustrated in earlier work.^15,16^

**Figure 1:**
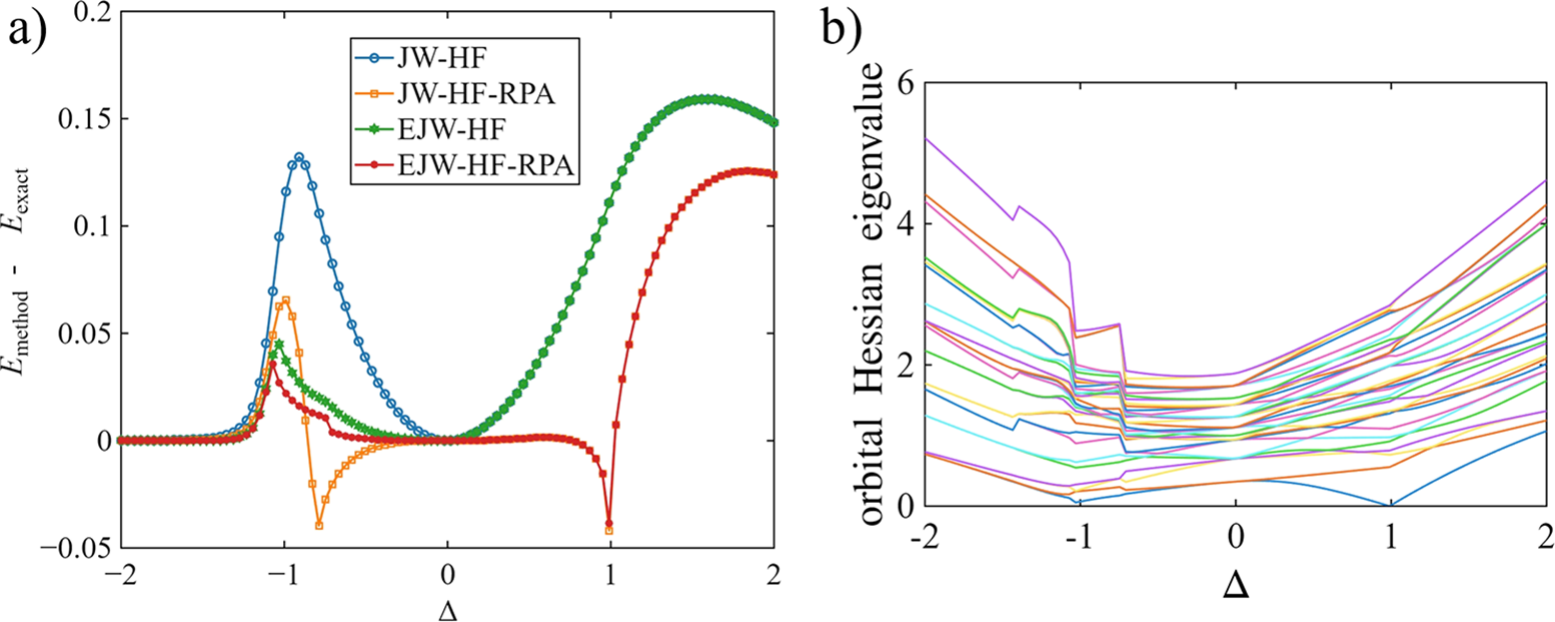
(a) Ground-state energy errors (in units of the uniform nearest-neighbor coupling, *J* = 1) for an open *N* = 8 spin-1/2 *XXZ* chain as a function of the anisotropy Δ. Shown are the results for HF (with sequential numbering of sites) and oo-uLAST. (b) Spectrum of the orbital Hessian for oo-uLAST.

Figure 2 shows the performance of oo-uLAST and RPA in terms of the relative ground-state energy error, (*E*_exact_ – *E*_method_)/*E*_exact_, for a family of systems with 24 spin-1/2 sites arranged in increasingly compact geometries: from a one-dimensional chain to quasi-2D ladders (12 × 2, 8 × 3) and the more compact 6 × 4 layout, each considered with open and periodic boundary conditions (OBC/PBC). Across all geometries and boundary conditions, the RPA correlation reduces the energy error over essentially the entire Δ range. The improvement is particularly pronounced in regions where the oo-uLAST reference is least accurate—most visibly near the sharp feature around Δ ≈ –1 and in the broad maximum at positive Δ—and it persists as the connectivity becomes more two-dimensional. At the same time, the residual errors increase as the arrangement becomes more compact and when periodic boundary conditions are imposed. These results can be compared to our earlier LAST-based correlation treatment on the same set of systems.^16^ While LAST typically achieves smaller absolute errors than RPA when it converges reliably, the present data show that RPA already captures a substantial fraction of the missing correlation at comparatively low additional cost and without the iterative amplitude optimization required by LAST. In this sense, oo-uLAST+RPA provides an efficient and robust correlation layer that complements the more demanding LAST treatment.

**Figure 2:**
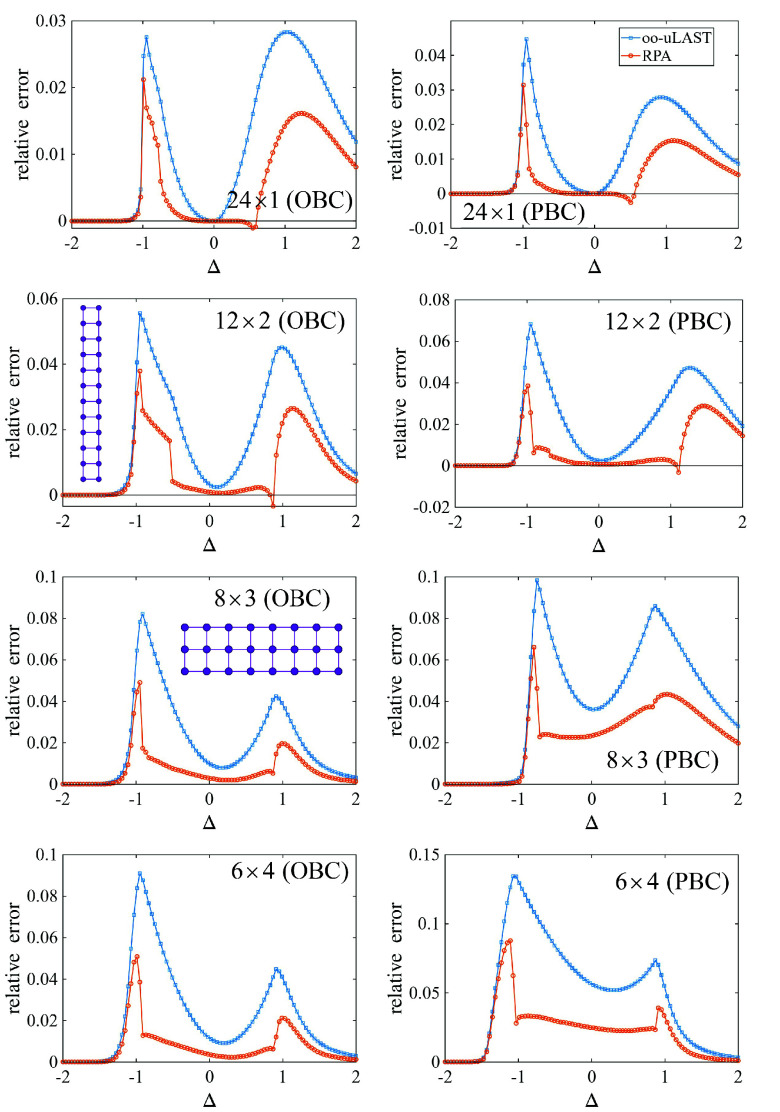
Relative ground-state energy errors for the spin-1/2 *XXZ* model on 24-site lattices as a function of Δ.

The oo-uLAST results shown in Figure 2 are identical to those reported in our earlier work,^16^ and we have verified that the same solutions can be reproduced using the SCF algorithm introduced in the present work. For all systems, we verified that the final mean-field references are HF-stable according to the orbital-Hessian criterion. We attribute this in part to our protocol for selecting low-energy mean-field solutions along parameter scans:^16^ for each Δ, we converge multiple oo-uLAST optimizations from random initial guesses and then propagate solutions stepwise by seeding neighboring Δ values, retaining the lowest-energy solution at each point. Since JW-HF/oo-uLAST commonly admits several energetically close stationary solutions, this strategy increases the likelihood of tracking the lowest-energy (and typically stable) branch rather than converging to a nearby saddle point. Finally, note that different local optimization procedures for the HF problem (e.g., gradient-based minimization in Thouless parameters versus SCF iterations) can have different basins of attraction and therefore may converge to different stationary solutions, even when started from the same initial guess.

### 3.2 *s* > 1/2

To illustrate how the approach performs beyond the spin-1/2 case, we consider an *N* = 4 *XXZ* ring with increasing on-site spin *s*. For $s>\frac{1}{2}$ , the EJW fermionization relies on auxiliary spin-1/2 degrees of freedom and therefore introduces a nontrivial string structure even in one-dimensional systems. As a result, the mean-field description is not trivially exact in special limits (in contrast to the spin-1/2 case at Δ = 0).

Figure 3 shows the relative ground-state energy error for oo-uLAST and RPA as a function of Δ in an *N* = 4 ring of *s* = 1 sites. The accuracy of oo-uLAST and its RPA correction correlate with the stability diagnostics provided by the orbital-Hessian spectrum. Over most of the scans, the RPA correction lowers the error substantially. A notable exception is the narrow interval around Δ ≈ 1, where both curves exhibit a sharp feature. Figure 3b helps to rationalize this behavior: In the same region, the lowest orbital-Hessian eigenvalue approaches zero, indicating that the mean-field solution is soft with respect to orbital rotations. In addition, the Hessian eigenvalues show a kink, consistent with a near-degeneracy between mean-field solutions. Away from this narrow region, most clearly toward larger positive Δ, both the mean-field and RPA-corrected energies approach the exact result as the ground state becomes increasingly classical in the eas*y*-axis limit. Note that, even though the mean-field reference breaks the underlying *S*_*x*_ and *S*_*y*_ spin-rotation symmetry at the isotropic point, this does not produce orbital-Hessian zero modes (cf. Figure 3b), because rotations generated by *S*_*x*_ and *S*_*y*_ are not one-body symmetries in the JW/EJW fermionic representation (and they mix particle-number sectors).

**Figure 3:**
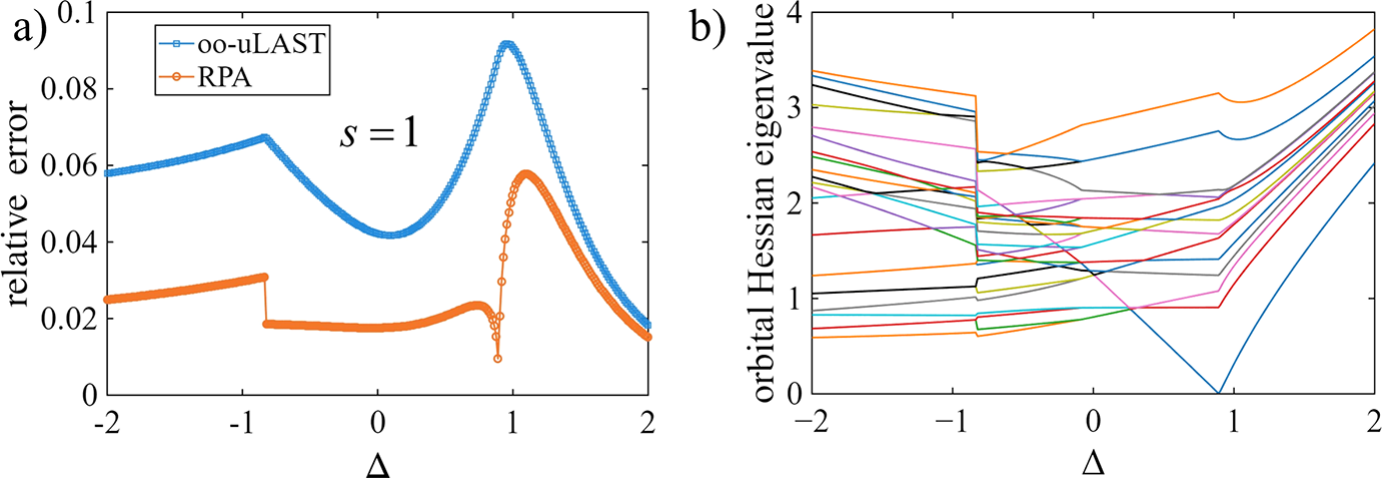
Relation between energetics and mean-field stability diagnostics for an *N* = 4 spin-1 ring. (a) Relative ground-state energy errors versus Δ for oo-uLAST and oo-uLAST+RPA. (b) Orbital-Hessian eigenvalues of the oo-uLAST references. The softening of a mode near Δ ≈ 1 indicates a change in the mean-field reference.

Figure 4 extends the *N* = 4 *XXZ* ring benchmarks to on-site spins $s=\frac{3}{2},2,\frac{5}{2},3$ , where the same qualitative pattern emerges: The RPA step provides a systematic reduction of the mean-field error over a broad parameter range. A common feature is a sharp structure in the vicinity of Δ ≈ 1, which coincides with a change in the character of the mean-field solution along the scan (in all cases, a single orbital-Hessian eigenvalue approaches zero; data not shown).

**Figure 4:**
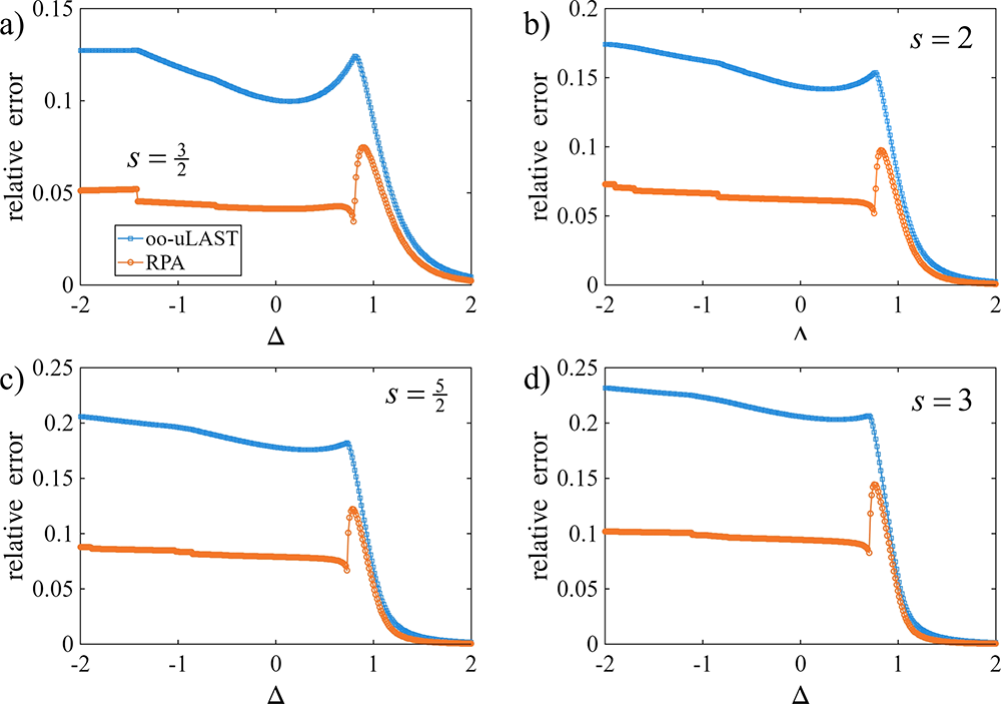
Relative energy errors for an *XXZ* ring with on-site spins (a) $s=\frac{3}{2}$ , (b) *s* = 2, (c) $s=\frac{5}{2}$ , and (d) *s* = 3.

### 3.3 *J*_1_–*J*_2_ Model

Figure 5 benchmarks the isotropic antiferromagnetic *J*_1_–*J*_2_ model at fixed size *N* = 24 for the same geometries as in Figure 2 as a function of the relative magnitude of the frustration-inducing NNN coupling *J*_2_. Across all geometries and parameter values 0 ≤ *J*_2_/*J*_1_ ≤ 1, the oo-uLAST reference (same as in ref 16) yields a relative energy error <10%, with several systems displaying zero error at the Majumdar–Ghosh point *J*_2_/*J*_1_ = 0.5 due to dimer formation.^40^ For all systems, the RPA correction yields a systematic error reduction. The gain is especially evident in the 12 × 2 PBC lattice with errors <1% over a large fraction of the scan region. At the same time, the RPA curves exhibit localized sharp features (spikes, kinks) and occasional sign changes of the error (overcorrelation) in narrow windows, most prominently around *J*_2_/*J*_1_ ≈ 0.6 for several OBC geometries and around *J*_2_/*J*_1_ ≈ 0.9 in some chain/PBC cases, again signaling changes of the optimized mean-field reference, which makes any response-based correction sensitive to small parameter changes. However, the magnitude of the oo-uLAST+RPA error remains substantially below the underlying oo-uLAST reference in most cases. Overall, PBC and more compact clusters generally show larger residual errors, consistent with the increased correlation demands for a larger number of closed loops. The spikes and kinks in the RPA error curves have a well-defined physical origin: They arise at parameter values where competing Hartree–Fock solutions become nearly degenerate, and the lowest-energy solution undergoes a qualitative change. This is directly analogous to level crossings in electronic-structure theory. Such features correspond loosely to the existence of multiple phases in the thermodynamic limit and are a well-known consequence of finite-size mean-field calculations near phase boundaries. In practice, the orbital-Hessian spectrum serves as a built-in diagnostic: A low-lying eigenvalue approaching zero flags the region where the RPA results should be interpreted with caution. These features are confined to narrow parameter windows and do not compromise the overall accuracy of the method across the broad parameter regimes studied.

**Figure 5:**
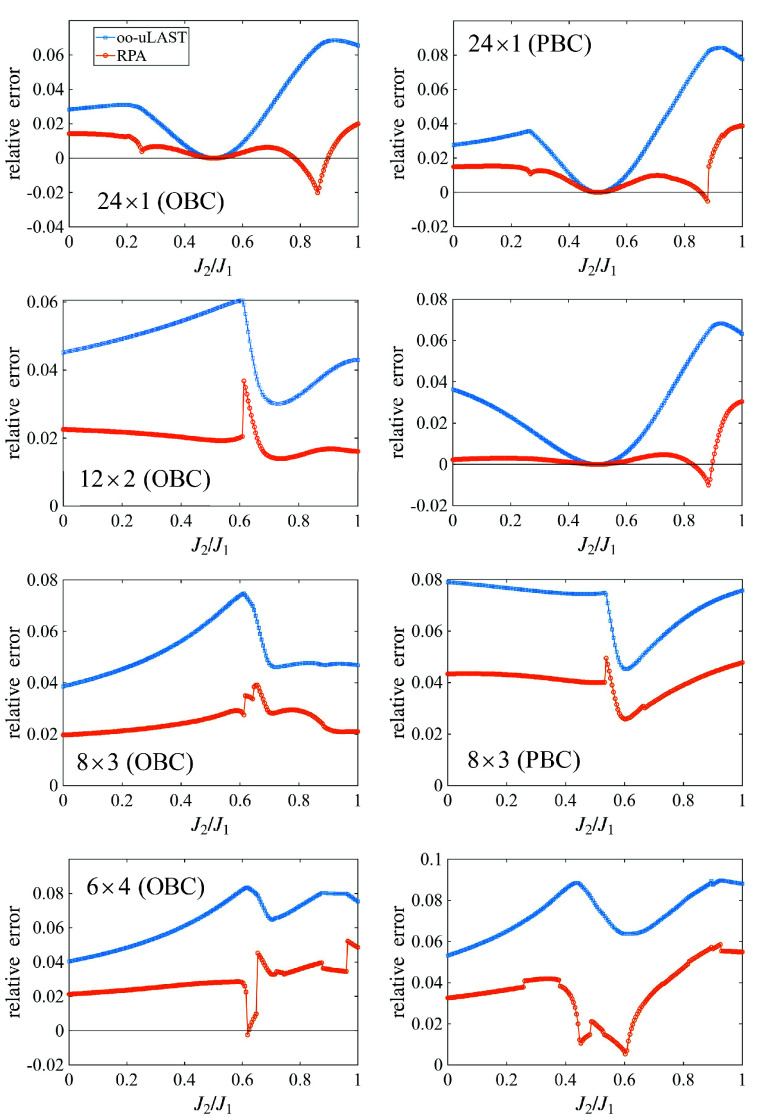
Relative energy errors for the isotropic antiferromagnetic *J*_1_–*J*_2_ Heisenberg model for lattices with 24 spin-1/2 sites (same geometries as in Figure 2).

## 4 Summary and Conclusions

In this work, we developed a practical self-consistent field (SCF) and correlation framework for spin Hamiltonians in the fermionic extended Jordan–Wigner (EJW) representation. While mean-field optimization on general coupling topologies can be carried out via Thouless parameters,^16^ the complementary SCF formulation, casting the HF energy as a functional of the single-particle density matrix, provides a convenient route to established SCF convergence techniques^18^ and a natural interface to response theory machinery on top of oo-uLAST references, where the free parameters of the EJW transformation and the mean-field state are optimized simultaneously. We note that the formalism is specifically designed for arbitrary (non-one-dimensional) coupling topologies, where the Jordan–Wigner strings do not reduce to the identity and standard fermionic methods cannot be applied directly.

We derived the analytical orbital Hessian for EJW-transformed spin Hamiltonians and implemented it as a stability diagnostic for stationary HF solutions. The same building blocks define the corresponding RPA matrix, enabling us to compute ground-state correlation energies (here in the exchange-including ring-CCD convention^25^) at moderate additional cost.

Benchmarks for the *XXZ* model and the antiferromagnetic *J*_1_−*J*_2_ model on one- and two-dimensional geometries show that adding the RPA correlation yields a systematic improvement over the underlying mean-field energetics across most parameter regimes. Residual errors tend to increase for more compact clusters and under periodic boundary conditions, consistent with higher correlation demands. Overcorrelation by RPA may occur in narrow windows and is typically indicated by a softening of low-lying orbital-Hessian modes due to competing (near-degenerate) mean-field solutions.

Overall, the SCF formulation together with orbital-Hessian diagnostics and an RPA correlation step provides a scalable route to approximate ground-state energies of JW-transformed spin models on arbitrary graphs and continues a line of work that we consider worthy of further exploration: For spin Hamiltonians, which represent paradigmatic models of strong correlation, fermionic mean-field descriptions based on the JW/EJW transformation combined with systematically improvable correlation treatments can offer efficient and accurate approximations. We note that the orbital Hessian derived here also provides the building blocks for the coupled-perturbed Hartree–Fock equations, enabling the computation of response properties in future work. Furthermore, while RPA already provides a substantial and computationally inexpensive improvement over the mean-field description, it represents a first rung on the correlation ladder. Exploring more sophisticated correlation methods in the fermionic JW/EJW frame—bearing in mind that the nonlocal string structure of the Hamiltonian may require adaptations beyond standard coupled-cluster formulations—is a natural direction for future work.

## Appendix A

### A.1 Formulation of the uLAST Gradient as a Density Matrix Functional

In our earlier work,^16^ for the optimization of **θ** defining the EJW transformation (see Section 2), we evaluated the gradient based on the Hellmann–Feynman theorem (eq 42),

$$\vartheta_{kl}\equiv \frac{\partial \left\langle \Phi \right|H\left(\theta \right)\left|\Phi \right\rangle }{\partial \theta_{kl}}=\left\langle \Phi \right|\frac{\partial H\left(\theta \right)}{\partial \theta_{kl}}\left|\Phi \right\rangle$$
42

by computing the expectation value of the derivative of the EJW-transformed Hamiltonian *H*(**θ**) with respect to the HF wave function. As an alternative, we here derive a gradient expression that refers only to the density matrix **ρ** instead of the Slater determinant. This formulation would enable an approach akin to thermal HF, i.e., thermal oo-uLAST. When using the gradient developed here, the oo-uLAST method consists of a nested iterative procedure: an HF routine to obtain a stationary mean-field state followed by gradient-based optimization of the uLAST parameters. Note that the SCF micro-iterations are not required to be solved to a fixed tolerance at all macro-iterations. Instead, one can tighten the SCF stationarity threshold as the optimization proceeds when the uLAST parameters are still far from optimal, while ensuring that the uLAST gradient (derived here for stationary mean-field states) is evaluated in its regime of validity.

The energy gradient, according to eq 43, consists of a fixed-density term and a contribution for the coupling with density variations. All partial derivatives are understood to fix the other independent θ_*pq*_ angles. We use the notation ⟨**A**, **B**⟩ ≡ Tr­(**A** ^†^ **B**) for the Frobenius product of two matrices in eq 43.

$$\frac{\partial E}{\partial \theta_{kl}}={\left(\frac{\partial E}{\partial \theta_{kl}}\right)}_{\rho }+\left\langle \frac{\delta E}{\delta \rho },\frac{\partial \rho }{\partial \theta_{kl}}\right\rangle$$
43

For a self-consistent solution, with [**F**, **ρ**] = 0, δ*E*/δ**ρ** = 0, eq 43 simplifies to eq 44:

$$\frac{\partial E}{\partial \theta_{kl}}={\left(\frac{\partial E}{\partial \theta_{kl}}\right)}_{\rho }$$
44

As in the derivation leading to eq 28 above, we consider the energy contribution from *xy*-coupling for an ordered pair in eq 45:

$$\varepsilon =w\mathrm{Tr}\left({h\rho }_{R}\right)=w\mathrm{Tr}\left({\mathrm{hRZ}}^{-1}\rho \right)$$
45

**R** represents the diagonal matrix of eq 46. The relation between {α_*i*_} and the parameters **θ** will be established in eq 54 below.

$$R=\left(\begin{array}{ccc} e^{i\alpha_{1}} & 0 & 0\\ 0 & \ddots & 0\\ 0 & 0 & e^{i\alpha_{N_{\mathrm{orb}}}} \end{array}\right)$$
46

Our aim is to compute eq 47, with **ρ** fixed:

$$\frac{\partial \varepsilon }{\partial \alpha_{q}}=\frac{\partial w}{\partial \alpha_{q}}\mathrm{Tr}\left({h\rho }_{R}\right)+w\mathrm{Tr}\left(h\frac{\partial \rho_{R}}{\partial \alpha_{q}}\right)$$
47

In eq 48, **E**_*qq*_ = **e**_*q*_ **e**_*q*_^*T*^ is a projector along *q*, with **e** *_q_* being the respective unit vector.

$$D_{q}\equiv \frac{\partial R}{\partial \alpha_{q}}=iE_{qq}R$$
48

The consecutive steps in eq 49 use (i) the Jacobi formula, (ii) eq 48, (iii) the cyclicity of the trace:

$$\frac{\partial w}{\partial \alpha_{q}}=w\mathrm{Tr}(Z^{-1}\rho D_{q})=iw\mathrm{Tr}(Z^{-1}\rho E_{q}R)=iw\mathrm{Tr}(E_{q\;}\rho_{R})=iw(\rho_{R}{)}_{qq}$$
49

For the derivative of **ρ** *_R_*, we need eqs 50 and 51:

$$\frac{\partial Z}{\partial \alpha_{q}}={\rho D}_{q}$$
50

$$\frac{\partial Z^{-1}}{\partial \alpha_{q}}=-Z^{-1}{\rho D}_{q}Z^{-1}$$
51

Combining eqs 21 and 49 then yields eq 52:

$$\frac{\partial \rho_{R}}{\partial \alpha_{q}}=D_{q}Z^{-1}\rho -{\mathrm{RZ}}^{-1}{\rho D}_{q}Z^{-1}\rho$$
52

The gradient (for fixed **ρ**) (eq 53),

$$g_{q}\equiv \frac{\partial E}{\partial \alpha_{q}}=iw\left[{\left(\rho_{R}\right)}_{qq}\mathrm{Tr}\left({h\rho }_{R}\right)+{\left(\rho_{R}h\right)}_{qq}-{\left(\rho_{R}{h\rho }_{R}\right)}_{qq}\right]$$
53

can be formulated as a functional of **ρ**, based on eqs 21 and 14.

Reintroducing the *mn* labels, i.e., ε_*mn*_ = *w*_*mn*_Tr­(**h**_*mn*_ **ρ**_*R*_^*mn*^), with ${\left(h_{mn}\right)}_{ij}=\frac{1}{2}J_{mn}\delta_{im}\delta_{jn}$ and |Φ_*R*_⟩ = ϕ_*m*(*n*)_^†^ϕ_*n*(*m*)_|Φ⟩, the angles in **R** (cf. eq 46) are given by eq 54:

$$\alpha_{q}=\left(\theta_{mq}-\theta_{nq}\right)\left(1-\delta_{mq}\right)\left(1-\delta_{nq}\right)$$
54

Employing the chain rule, we get eq 55 for the independent parameters θ_*kl*_ (*k* < *l*).

$$\frac{\partial \varepsilon_{mn}}{\partial \theta_{kl}}=\underset{q}{\Sigma }g_{q}\frac{\partial \alpha_{q}}{\partial \theta_{kl}}=\underset{q}{\Sigma }g_{q}\frac{\partial (\delta_{mq}-\delta_{nq})}{\partial \delta_{kl}}(1-\delta_{mq})(1-\delta_{nq})=\underset{q}{\Sigma }g_{q}[(\delta_{mk}\delta_{ql}+\delta_{ml}\delta_{qk})-(\delta_{nk}\delta_{ql}+\delta_{nl}\delta_{qk})]\times (1-\delta_{mq})(1-\delta_{nq})=g_{l}(\delta_{mk}-\delta_{nk})(1-\delta_{ml})(1-\delta_{nl})+g_{k}(\delta_{ml}-\delta_{nl})(1-\delta_{mk})(1-\delta_{nk})$$
55

As *g*_*m*_ = *g*_*n*_ = 0, this can be simplified (eq 56):

$$\frac{\partial \varepsilon_{mn}}{\partial \theta_{kl}}=g_{l}\left(\delta_{mk}-\delta_{nk}\right)+g_{k}\left(\delta_{ml}-\delta_{nl}\right)$$
56

The total gradient is a sum over all ordered pairs (eq 57):

$$\frac{\partial E}{\partial \theta_{kl}}=\underset{m\neq n}{\sum }\frac{\partial \varepsilon_{mn}}{\partial \theta_{kl}}$$
57

### A.2 Derivation of the Orbital Hessian

A stationary point (HF solution) **ρ**_0_ satisfies [**F**_0_, **ρ**_0_], with **F**_0_ ≡ **F**[**ρ**_0_]. We work in the canonical MO basis, where **F**_0_ and **ρ**_0_ are diagonal, i.e., **F**_0_ = diag ({ε_*p*_}) ≡ **ε** (orbital energies are sorted in ascending order) and **ρ**_0_ = diag (**1**_*N*_*o*__, **0**_*N*_*v*__); *N*_*o*_ and *N*_*v*_ are the numbers of occupied and virtual orbitals, respectively. According to the Thouless theorem,^19^ every non-orthogonal Slater determinant—and thus every nearby Slater determinant—results from an occupied-virtual unitary transformation (Thouless rotation) of **ρ**_0_ (eq 58),

$$\rho =e^{t\kappa }\rho_{0}e^{-t\kappa }$$
58

where *t* is a real scaling parameter, and the anti-Hermitian **κ** = −**κ** ^†^ (eq 59),

$$\kappa =T-T^{†}$$
59

is defined in terms of a matrix **T** (eq 60),

$$T=\left(\begin{array}{cc} 0 & 0\\ Z & 0 \end{array}\right)$$
60

comprising occupied-virtual transition amplitudes (Thouless parameters), $Z\in C^{N_{v}\times N_{o}}$ . In operator form (eq 61),

$$T=\underset{vo}{\sum }Z_{vo}a_{v}^{†}a_{o}$$
61

*a*_*p*_^†^ (*a*_*p*_) creates (annihilates) a fermion in the *p*-th MO (see eq 62):

$$a_{p}^{†}=\underset{q}{\sum }C_{qp}c_{q}^{†}$$
62

In the Taylor expansion of eq 63,

$$\rho \left(t\right)=\rho_{0}+\rho_{0}^{\prime }t+\frac{1}{2}\rho_{0}^{\prime \prime }t^{2}+O\left(t^{3}\right)$$
63

the first- and second-order derivatives, eqs 64 and ([Disp-formula eq65]), result from the Baker–Campbell–Hausdorff expansion.

$$\frac{d\rho }{dt}{|}_{t=0}\equiv \rho_{0}^{\prime }=[\kappa ,\rho_{0}]=\left(\begin{array}{cc} 0 & Z^{†}\\ Z & 0 \end{array}\right)$$
64

$$\rho_{0}^{\prime \prime }=\left[\kappa ,\left[\kappa ,\rho_{0}\right]\right]=2\left(\begin{array}{cc} -Z^{†}Z & 0\\ 0 & {\mathrm{ZZ}}^{†} \end{array}\right)$$
65

In the second-order expansion of the HF energy, with *E*_0_ ≡ *E*[**ρ**_0_] (eq 66),

$$E\left[\rho \right]=E_{0}+E_{0}^{\prime }t+\frac{1}{2}E_{0}^{\prime \prime }t^{2}+O\left(t^{3}\right)$$
66

the first derivative (eq 67),

$$E^{\prime }=\mathrm{Tr}\left(F\rho^{\prime }\right)$$
67

vanishes at the stationary point (eq 68):

$$E_{0}^{\prime }=\mathrm{Tr}(F_{0}\;\rho_{0}^{\prime })=\mathrm{Tr}(F_{0}[\kappa ,\rho_{0}])=\mathrm{Tr}([F_{0},\rho_{0}]\kappa )=0$$
68

The second derivative is given by eq 69:

$$E^{\prime \prime }=\mathrm{Tr}\left(F^{\prime }\rho^{\prime }\right)+\mathrm{Tr}\left(F\rho^{\prime \prime }\right)$$
69

Through the chain rule, the response of the Fock matrix to a density variation (eq 70),

$$F_{pq}^{\prime }=\underset{rs}{\sum }K_{pqrs\;}\rho_{rs}^{\prime }$$
70

is encoded in the kernel **K** (eq 71),

$$K_{pqrs}\equiv \frac{\partial F_{pq}}{\partial \rho_{rs}}$$
71

The first term in eq 69 can thereby be formulated as eq 72:

$$\mathrm{Tr}\left(F^{\prime }\rho^{\prime }\right)=\underset{pqrs}{\sum }K_{pqrs}\rho_{rs}^{\prime }\rho_{qp}^{\prime }$$
72

By the definition of the Fock matrix, *F*_*pq*_ = ∂*E*/∂ρ_*qp*_, the kernel collects the second energy derivatives, *K*_*pqrs*_ = ∂^2^ *E*/∂ρ_*qp*_∂ρ_*rs*_ (note the order of indices in our convention). A derivation of **K** is provided in Appendix A3.

The second term of eq 69 is evaluated at the HF point in eq 73, using the fact that **F**_0_^*vv*^ = **ε**_*v*_ and **F**_0_^*oo*^ = **ε**_*o*_ are diagonal.

$$\mathrm{Tr}\left[F_{0\;}\rho_{0}^{\prime \prime }\right]=2\left[\mathrm{Tr}\left(F_{0}^{vv}{\mathrm{ZZ}}^{†}\right)-\mathrm{Tr}\left(F_{0}^{oo}Z^{†}Z\right)\right]=2\underset{vo}{\sum }\left(\varepsilon_{v}-\varepsilon_{o}\right){\left|Z_{vo}\right|}^{2}$$
73

Overall, the second energy derivative can be formulated as eq 74:

$$E_{0}^{\prime \prime }=\underset{pqrs}{\sum }K_{pqrs}{\left(\rho_{0}^{\prime }\right)}_{rs}{\left(\rho_{0}^{\prime }\right)}_{qp}+2\underset{vo}{\sum }\left(\varepsilon_{v}-\varepsilon_{o}\right){\left|Z_{vo}\right|}^{2}$$
74

By vectorizing **Z** as $z=\mathrm{vec}\left(Z\right)\in C^{N_{v}N_{o}}$ in column-major convention (*z*_*v*+(*o*–1)*N*_*v*__ = *Z*_*vo*_; Matlab code: *z* = *Z*(:)), the orbital-gap term can be reformulated as in eq 75,

$$\underset{v,o}{\sum }\left(\varepsilon_{v}-\varepsilon_{o}\right){\left|Z_{vo}\right|}^{2}=z^{†}\cdot A^{\Delta }\cdot z$$
75

with eq 76,

$$A^{\Delta }=1_{N_{o}}⊗\varepsilon_{v}-\varepsilon_{o}⊗1_{N_{v}}$$
76

or, in component notation:

$$A_{ai,bj}^{\Delta }=\left(\varepsilon_{a}-\varepsilon_{i}\right)\delta_{ab}\delta_{ij}$$
77

In eq 77 and hereafter in this section, indices *a*, *b* and *i*, *j* are used for virtual and occupied MOs, respectively, and *p*, *q*, *r*, and *s* are used for arbitrary MOs.

With **ρ**_0_^′^ having only *vo* and *ov* blocks, cf. eq 64, (**ρ**_0_^′^)_*ai*_ = *Z*_*ai*_, (**ρ**_0_^′^)_*ia*_ = *Z*_*ai*_^*^, we reformulate the kernel term in eq 78:

$$\underset{pqrs}{\sum }K_{pqrs}{\left(\rho_{0}^{\prime }\right)}_{rs}{\left(\rho_{0}^{\prime }\right)}_{qp}=\underset{\underset{\left(A\right)}{︸}}{\underset{abij}{\sum }K_{jbai}Z_{ai}Z_{bj}}+\underset{\underset{\left(B\right)}{︸}}{\underset{abij}{\sum }K_{bjai}Z_{ai}Z_{bj}^{*}}+\underset{\underset{\left(C\right)}{︸}}{\underset{abij}{\sum }K_{jbia}Z_{ai}^{*}Z_{bj}}+\underset{\underset{\left(D\right)}{︸}}{\underset{abij}{\sum }K_{bjia}Z_{ai}^{*}Z_{bj}^{*}}$$
78

Using the general relations *K*_*pqrs*_ = *K*_*rspq*_^*^ and *K*_*pqrs*_ = *K*_*qpsr*_^*^, we have *K*_*jbia*_ = *K*_*aibj*_ in term (*C*). Renaming indices (*i*, *a*) ↔ (*j*, *b*) in term (*B*), *K*_*bjai*_ *Z*_*ai*_ *Z*_*bj*_^*^ → *K*_*aibj*_ *Z*_*bj*_ *Z*_*ai*_^*^ then shows that (*B*) = (*C*). Similarly, we have (*A*) = (*D*)*. Thus, by vectorizing **Z** as **z**, we can write eq 79,

$$\underset{pqrs}{\sum }K_{pqrs}{\left(\rho_{0}^{\prime }\right)}_{rs}{\left(\rho_{0}^{\prime }\right)}_{qp}=2z^{†}\cdot A^{K}\cdot z+\left(z^{†}\cdot B\cdot z^{*}+c.c.\right)$$
79

with **A** *^K^* and **B** defined in component form in eqs 80 and 81:

$$A_{ai,bj}^{K}=K_{aibj}$$
80

$$B_{ai,bj}=K_{aijb}$$
81

Putting this all together, we get eq 82:

$$E_{0}^{\prime \prime }=2z^{†}\cdot \left(A^{K}+A^{\Delta }\right)\cdot z+\left(z^{†}\cdot B\cdot z^{*}+c.c.\right)$$
82

Stacking the amplitudes and their conjugates into the vector **u** in eq 83,

$$u=\left(\begin{array}{c} z\\ z^{*} \end{array}\right)$$
83

affords the standard orbital-Hessian block form (eq 84),

$$E_{0}^{\prime \prime }=u^{†}\cdot \left(\begin{array}{cc} A & B\\ B^{*} & A^{*} \end{array}\right)\cdot u=u^{†}\cdot H\cdot u$$
84

where **A** = **A** *^K^* + **A** ^Δ^. The analytic expression for the orbital Hessian was validated against numerical Hessians obtained from a Thouless-parameter optimization of the Slater determinant (see ref 16), using the fminunc function in the MATLAB Optimization Toolbox.

### A.3 Fock Kernel

As explained in the main text, to assemble the particle-hole response matrices **A** and **B**, we need the Fock kernel, *K*_*pqrs*_ ≡ ∂*F*_*pq*_/∂ρ_*rs*_. We first examine the *xy*-contribution, **K** *^xy^*, followed by the simpler *z*-part, **K** *^z^*, and finally combine both to obtain the total kernel, **K** = **K** *^xy^* + **K** *^z^*. Starting from the scalar Fock increment **f** for the *xy*-contribution of an ordered pair from eq 28, we take the total differential (at fixed uLAST parameters) in eq 85.

$$df=dw[{\mathrm{hRZ}}^{-1}-(R-1)Z^{-1}{\rho \mathrm{hRZ}}^{-1}+\mathrm{Tr}({\mathrm{hRZ}}^{-1}\rho )(R-1)Z^{-1}]+w[\mathrm{hR}d(Z^{-1})-(R-1)d(Z^{-1})\rho \mathrm{hR}Z^{-1}-(R-1)\times Z^{-1}(d\rho )\mathrm{hR}Z^{-1}-(R-1)Z^{-1}\rho \mathrm{hR}d(Z^{-1})+\{\mathrm{Tr}[\mathrm{hR}d(Z^{-1})\rho ]+\mathrm{Tr}[\mathrm{hR}Z^{-1}(d\rho )]\}(R-1)Z^{-1}+\mathrm{Tr}(\mathrm{hR}Z^{-1}\rho )(R-1)d(Z^{-1})]$$
85

In eq 86, we provide the derivative κ_*pqrs*_ ≡ ∂*f*_*pq*_/∂ρ_*rs*_, keeping terms in the same order as in eq 85.

$$\kappa_{pqrs}=\frac{\partial w}{\partial \rho_{rs}}\times {\left[{\mathrm{hRZ}}^{-1}-\left(R-1\right)Z^{-1}{\rho \mathrm{hRZ}}^{-1}+\mathrm{Tr}\left({\mathrm{hRZ}}^{-1}\rho \right)\left(R-1\right)Z^{-1}\right]}_{pq}+w\left\{{\left[\mathrm{hR}\frac{\partial \left(Z^{-1}\right)}{\partial \rho_{rs}}\right]}_{pq}-{\left[\left(R-1\right)\frac{\partial \left(Z^{-1}\right)}{\partial \rho_{rs}}{\rho \mathrm{hRZ}}^{-1}\right]}_{pq}-{\left[\left(R-1\right)Z^{-1}\right]}_{pr}{\left[{\mathrm{hRZ}}^{-1}\right]}_{sq}-{\left[\left(R-1\right)Z^{-1}\rho \mathrm{hR}\frac{\partial \left(Z^{-1}\right)}{\partial \rho_{rs}}\right]}_{pq}+\left[\mathrm{Tr}\left(\mathrm{hR}\frac{\partial \left(Z^{-1}\right)}{\partial \rho_{rs}}\rho \right)+{\left[{\mathrm{hRZ}}^{-1}\right]}_{sr}\right]{\left[\left(R-1\right)Z^{-1}\right]}_{pq}+\mathrm{Tr}({\mathrm{hRZ}}^{-1}\rho ){\left[\left(R-1\right)\frac{\partial \left(Z^{-1}\right)}{\partial \rho_{rs}}\right]}_{pq}\right\}$$
86

To proceed, we work out the derivatives appearing in eq 86. We begin with the overlap derivative in eq 87,

$$\frac{\partial w}{\partial \rho_{rs}}=w\mathrm{Tr}\left[\left(R-1\right)Z^{-1}E_{rs}\right]=w{\left[\left(R-1\right)Z^{-1}\right]}_{sr}$$
87

where (**E** *_rs_*)*_ij_* = δ_*ri*_δ_*sj*_ (in terms of unit vectors along *r* and *s*, **E**_*rs*_ = **e**_*r*_ **e**_*s*_^*T*^); the second equality uses the general relation Tr­(**ME** *_rs_*) = *M*_*sr*_. For **Z** ^–1^, we employ eq 24 to arrive at eq 88.

$$\begin{array}{l} \frac{\partial \left(Z^{-1}\right)}{\partial \rho_{rs}}=-Z^{-1}E_{rs}\left(R-1\right)Z^{-1}\\ \Leftrightarrow {\left(\frac{\partial \left(Z^{-1}\right)}{\partial \rho_{rs}}\right)}_{pq}=-{\left(Z^{-1}\right)}_{pr}{\left[\left(R-1\right)Z^{-1}\right]}_{sq} \end{array}$$
88

Substituting eqs 87 and 88 into eq 86 and using the definitions of eqs 89 and 90, yields eq 91.

$$Q=\left(R-1\right)Z^{-1}$$
89

$$V={\mathrm{hRZ}}^{-1}$$
90

$$\begin{array}{l} \kappa_{pqrs}=w\{Q_{sr}{\left[V-Q\;\rho V+\mathrm{Tr}\left(V\rho \right)Q\right]}_{pq}\\ -V_{pr}Q_{sq}-Q_{pr}V_{sq}+Q_{pr}{\left(Q\;\rho V\right)}_{sq}+{\left(Q\;\rho V\right)}_{pr}Q_{sq}\\ +\left[-{\left(Q\;\rho V\right)}_{sr}+V_{sr}\right]Q_{pq}-\mathrm{Tr}\left(V\rho \right)Q_{pr}Q_{sq}\} \end{array}$$
91

Equation 91 represents the kernel increment κ_*pqrs*_^*mn*^ for an ordered pair ⟨*m*, *n*⟩. Summing over all ordered pairs yields the full *xy*-contribution, eq 92:

$$K^{xy}=\underset{m\neq n}{\sum }\kappa^{mn}$$
92

The *z*-part, **K** *^z^*, is straightforward. From eqs 8 and 9, we obtain eq 93.

$$K_{k\ln m}^{z}=\frac{\partial F_{kl}^{z}}{\partial \rho_{nm}}=\left[kl|mn\right]$$
93

The overall kernel, **K** ^AO^ = **K** *^xy^* + **K** *^z^*, is first obtained in the “atomic orbital” (AO) basis (site basis) and then transformed into the MO basis. Let **C** = (**v**_1_, **v**_2_, ···, **v**_*N*_orb__) be the unitary matrix of canonical orbitals (AO-basis eigenvectors of **F**), with coefficients (**v** *_p_*)*_μ_* = *C*_μ*p*_. Using AO indices, μ, ν, λ, σ, and MO indices *p*, *q*, *r*, and *s*, the AO → MO transformation of the four-index kernel is given in eq 94:

$$K_{pqrs}^{\mathrm{MO}}=\underset{\mu \nu \lambda \sigma }{\sum }C_{\mu p}^{*}C_{\nu q}K_{\mu \nu \lambda \sigma }^{\mathrm{AO}}C_{\lambda r}C_{\sigma s}^{*}$$
94

In the main text, we drop the MO superscript. The AO → MO transformation preserves the index symmetries, i.e., *K*_*pqrs*_ = *K*_*rspq*_^*^ and *K*_*pqrs*_ = *K*_*qpsr*_^*^.
